# Epicardial access for VT ablation: analysis of two different puncture techniques, incidence of adhesions and complication management

**DOI:** 10.1007/s00392-020-01711-z

**Published:** 2020-07-27

**Authors:** Shibu Mathew, Sebastian Feickert, Thomas Fink, Andreas Rillig, Bruno Reissmann, Laura Rottner, Naotaka Hashiguchi, Peter Wohlmuth, Tilman Maurer, Christine Lemes, Andreas Metzner, Karl-Heinz Kuck, Feifan Ouyang

**Affiliations:** 1 Department of Cardiology, Asklepios Klinik St. Georg, Hamburg, Germany; 2 Department of Cardiology, University Hospital of Giessen, Klinikstrasse 33, 35392 Giessen, Germany; 3 University Heart Center Lübeck, Medical Clinic II, University Hospital Schleswig Holstein, Lübeck, Germany; 4 Asklepios Proresearch, Hamburg, Germany; 5 University Heart Center Hamburg, Department of Electrophysiology, University Hospital Hamburg Eppendorf, Hamburg, Germany; 6 Fuwai Hospital/National Centers of Cardiovascular Diseases, The Chinese Academy of Medical Sciences and National Center of Cardiovascular Diseases, Beijing, China

**Keywords:** Catheter ablation, Ventricular tachycardia, Safety, Anterior epicardial puncture, Epicardial ablation, Pericardial adhesions

## Abstract

**Introduction:**

Pericardial access for ablation of ventricular arrhythmias (VA) can be gained either by an anterior-oriented or inferior-oriented epicardial puncture under fluoroscopical guidance. We retrospectively sought to assess the safety of these two puncture techniques and the incidence of epicardial adhesions and introduce our algorithm for management of pericardial tamponade.

**Methods and results:**

In 211 patients (61.4 ± 15.6 years, 179 males; 84.8%) 271 epicardial ablation procedures of VA were performed using either an anterior- or inferior-oriented approach for epicardial access. Puncture-related complications were systematically analyzed. Furthermore, the incidence of adhesions was evaluated during first and repeated procedures.

A total of 34/271 (12.5%) major complications occurred and 23/271 (8.5%) were directly related to epicardial puncture. The incidence of puncture-related major complications in the anterior and inferior group was 4/82 (4.9%) and 19/189 (10.1%), respectively. Pericardial tamponade was the most common major complication (15/271; 5.5%). Collateral damages of adjacent structures such as liver, colon, gastric vessels and coronary arteries occurred in 6/189 (3.2%) patients and only within the inferior epicardial access group. Adhesions were documented in 19/211 (9%) patients during the first procedure and in 47.1% if patients had 2 or more procedures involving epicardial access.

**Conclusion:**

Anterior-oriented epicardial puncture shows an observed association to a reduced incidence of pericardial tamponades and overall puncture-related complications in epicardial ablation of VA. In cases of repeated epicardial access adhesions increase significantly and may lead to ablation failure.

## Introduction

Endocardial catheter ablation of ventricular arrhythmias (VA) has become a widely established treatment modality in recent time [1, 2]. Since the first report on percutaneous subxiphoid puncture of the epicardial space by Sosa et al. in patients with Chagas disease the worldwide number of epicardial procedures increased rapidly and the indication for these procedures has been extended for different entities of cardiomyopathies [3–6]. However, epicardial ablation of VA is still challenging and time-consuming. The incidence of reported complications during these procedures remain high and are mainly caused during epicardial puncture to gain access to the epicardial space [7, 8]. In most institutions the “dry” subxiphoid puncture to the epicardium has been performed aiming for the inferior epicardial space under fluoroscopical guidance in an anteroposterior (AP) view so far [3]. This method leads to an inferior puncture to access the epicardial space easily in most cases but bears the risk of injuries of adjacent structures and abdominal organs [9, 10]. The possibility of entering the anterior portion of the pericardial sac through a more shallow, horizontal subxiphoid puncture has been described more than a decade ago by Sosa et al. [11]. Nevertheless, the initially described posterior-oriented needle-in-needle access remained the technique of choice in subsequent studies [7], mainly due to the experience of easier mapping and ablation of the inferior and lateral walls of the ventricles, as well as the left ventricle [12].

To avoid collateral damages, a fluoroscopically guided access using a left anterior oblique (LAO) 90° projection to obtain an anterior access to the pericardial space has been implemented at different institutions, delineating an acceptable safety profile and achievement of complete and detailed mapping of both ventricles [13, 14]. The procedural safety and characteristics have not been analyzed systematically so far. Besides the challenge of a safe access to the epicardium, epicardial adhesions are a factor that may complicate these procedures [14, 15]. In this study, we sought to assess the safety outcome of these two different fluoroscopically guided puncture techniques, epicardial adhesions before and after epicardial procedures and the incidence of de novo adhesions, as well as a specific algorithm for the management of pericardial tamponades, the most common life-threatening complication of epicardial puncture.

## Methods

### Study design and patient inclusion

The study population consisted of 211 patients who were enrolled in our center between 2002 and 2017 with VA and received at least 1 epicardial ablation procedure. All patients had at least 1 symptomatic episode of sustained monomorphic VT documented by 12-lead ECG, Holter monitoring, or interrogation of the ICD [16].

The local ethical committee approved this retrospective study (processing number: WF-48/17). All patients included in the analysis gave written informed consent to the procedure, and patient information were anonymized for analysis.

### Procedural set up

Transesophageal echocardiography was performed to rule out intracardiac thrombi prior to the procedure in case of underlying atrial fibrillation (AF). In the case of preexisting treatment with a vitamin K antagonist, the procedure was performed on bridging therapy (INR < 2) with low-molecular heparin (LMH) and held > 6 h before the procedure. Novel oral anticoagulants (NOAC) were discontinued 24 h before the procedure, if medically acceptable. All interventions were performed under deep sedation using midazolam, sufentanyl and propofol. In patients with severely decompensated heart failure, hemodynamically instable VT/electrical storm, or major impairment of respiratory function procedures were performed under general anesthesia with endotracheal intubation.

If ECG criteria anticipated left ventricular access, retrograde transaortic and/or antegrade transseptal puncture was performed after epicardial puncture to avoid access under heparinization. Electrophysiological study, mapping and ablation were performed as described by our group previously [17].

### Epicardial procedures—inferior approach for epicardial access (group 1)

**Fig. 1:**
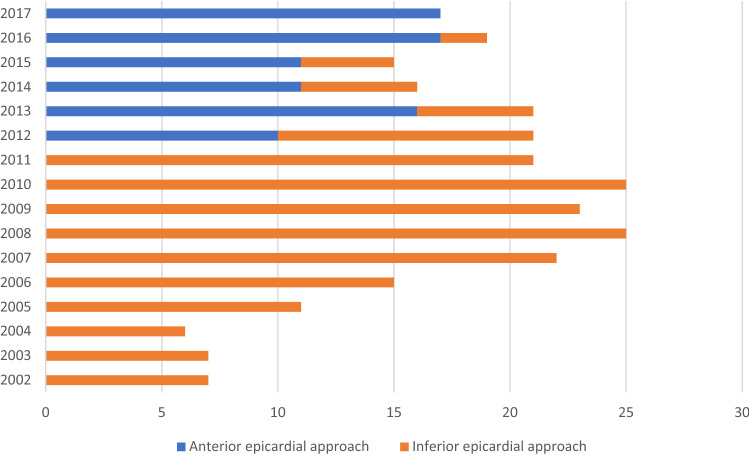
Proportion of anterior- and inferior oriented epicardial approaches over the years. *X*-axis indicates the total number of procedures with epicardial access. *Y*-axis indicates the year

**Fig. 2:**
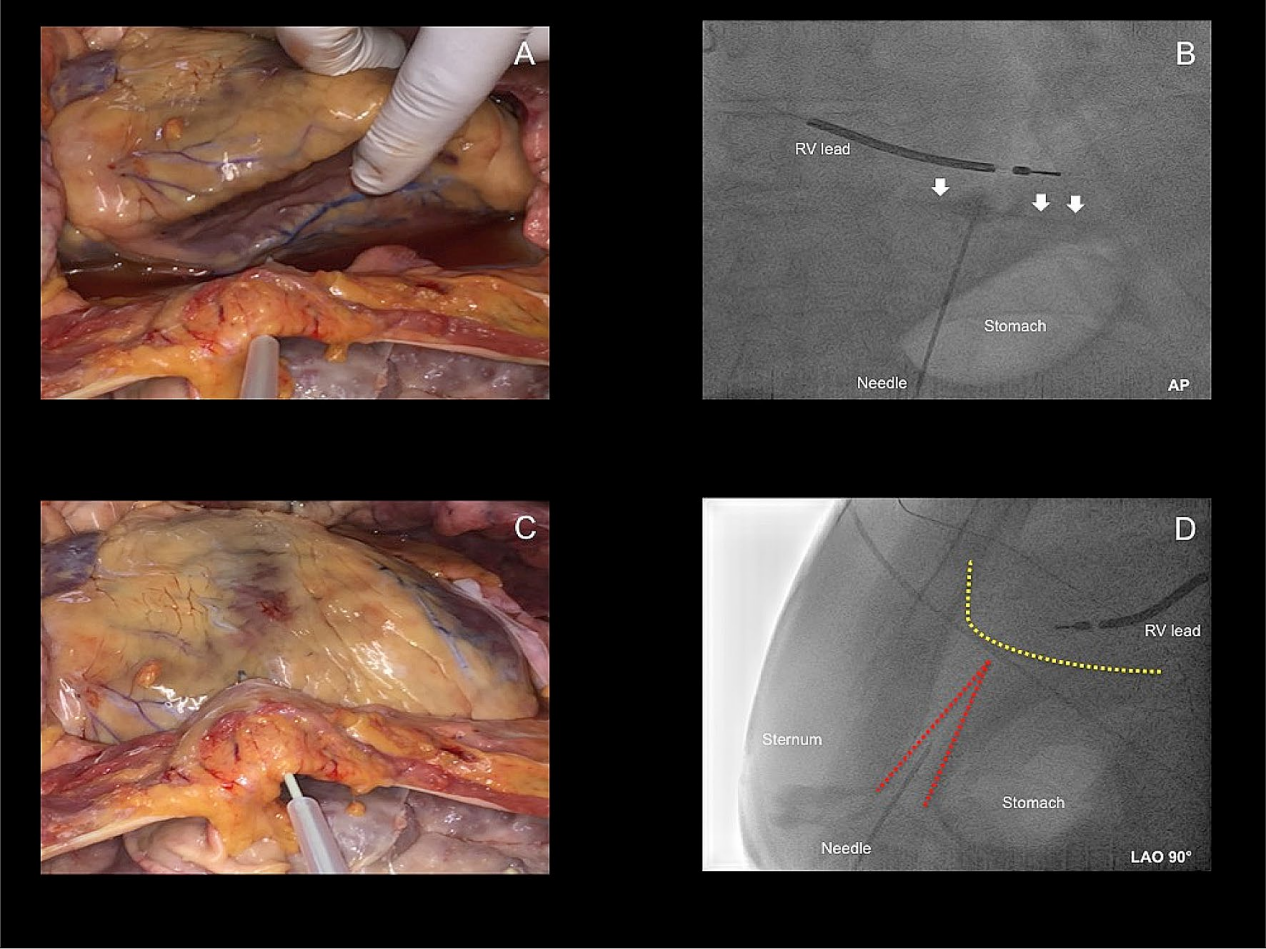
**a**, **b** Inferior epicardial access with fluoroscopy projection in AP. White arrows in **b** illustrate the contrast media within the pericardial space. **c**, **d** Anterior epicardial access with fluoroscopy projection in LAO 90°. Yellow dotted line illustrates the silhouette of the heart. Red dotted line illustrates the triangle between sternum, abdominal organs and heart. With permission of the Institute of Pathology, Asklepios Hospital St. George. *LAO* Left anterior oblique, *AP* Anteroposterior, *RV* right ventricle

Pericardial access was performed as previously described [17]. Until February 2012 the largest part of epicardial ablation procedures were regularly performed fluoroscopically guided in AP view with a 14-gauge cannula (Angiocath, Becton Dickinson, Sandy, UT, USA) in an inferior and left medioclavicular oriented direction, between the left border of the subxiphoid and the rib cage, the so-called Larrey’s gap (Figs. 1, 2). The needle was advanced in direction of the silhouette of the heart, demonstrated via an AP view. 2–5 milliL of contrast media were injected in order to visualize tenting of the pericardium. Under fluoroscopical guidance and tactile feedback a “dry” puncture of the fibrous pericardium was performed and resulted in the loss of resistance. Repeated injection of contrast media marked the pericardial space along with the silhouette of the heart (Fig. 2). After verification of the correct location of the needle a guidewire was advanced into the epicardial space projecting on multiple chambers of the heart to exclude accidental puncture of the right ventricle (RV) and stepwise dilatation of the puncture side of the skin with upsized dilatators (5F to 8F) was performed. Afterwards, a long 8.5F SL-1 sheath (Abott, St Paul, MN, USA) or a steerable sheath (Agilis, Abott, St Paul, MN, USA) was inserted over the guidewire into the epicardial space for mapping and ablation of the suspected arrhythmogenic substrate.

### Epicardial procedures—anterior approach for epicardial access (group 2)

The initially performed inferior-oriented epicardial access approach was modified and changed after February 2012 to an anterior-oriented puncture technique (Figs. 1, 2) due to a remaining high rate of cardiac and non-cardiac complications, as well as first reports about the safety profile of the anterior puncture technique [18, 19]. In this patient cohort, a “dry” subxiphoid puncture was performed under fluoroscopic guidance in AP and LAO 90° view. After initial puncturing of the skin below the xiphoid process, the needle was further maneuvered in a medioclavicular direction towards the pericardial space in LAO 90° guidance to distinguish the triangle of sternum, abdominal organs and right ventricle (Fig. 2d). The needle was slowly advanced directly below the sternum, but above the abdominal organs (Fig. 2d). Remaining steps of pericardial access were the same as in the inferior-oriented puncture group. Again, verification of the correct needle position was performed with contrast media and a long guidewire was advanced to the epicardial space. In addition, dilatation of the puncture side and advancement of a long 8.5F SL-1 or a steerable sheath were the final steps.

### Post-procedural care and in-hospital follow-up

At the end of the procedure, all residual fluid was aspirated from the epicardial space. Methylprednisolone (250 mg) was administered in all patients via the sheath into the epicardial space and the SL1 sheath was changed to a short 8F sheath. A pigtail drain was introduced into the pericardial space by Seldinger technique and remained inside the pericardial space in case of epicardial bleeding or serous fluid secretion. Patients in both groups underwent transthoracic echocardiography immediately after epicardial procedures as well as the next day. The epicardial pigtail catheter was removed after repeated exclusion of pericardial effusion via transthoracic echocardiography. In case of difficult subxiphoidal puncture, patients underwent chest X-ray and/or abdominal X-Ray to exclude pneumothorax or free abdominal air at the operator’s discretion. Anticoagulation was held on operator’s discretion as well, depending on the indication of anticoagulation and potential bleeding risk. Patient data were collected until discharge and all adverse events and deaths were documented.

### Definition of periprocedural complications

Procedural complications were defined to be major when being life-threatening or resulting in patient’s death, temporal or permanent patient disability, leading to interventional or surgical treatment, leading to transfusion of blood products or leading to prolonged hospital stay. All other complications were categorized to be minor.

### Complication and bleeding management

Since bleeding into the epicardial space was the most common complication in our patient population, we implemented a standardized complication management for cases of pericardial tamponade with an unexpectedly high blood loss, using always the same algorithm. First step was a drainage of blood either directly via the sheath or thorough a pigtail-catheter. If bleeding continued, an early autotransfusion as a secondary treatment step was performed. Drained blood from the epicardial space was autotransfused to a peripheral venous access via a filter system (Sangofix^®^, B. Braun, Melsungen AG, Germany) to stabilize patients during the initial phase and reduce the cumulative blood loss, which threatens to aggravate these situations. If previous steps did not lead to sufficient stabilization and pericardial effusion was still present and clinically relevant antagonisation of heparine through the administration of protamine was performed as a third step. After the reversal of anticoagulation, a combined attempt of drainage and flushing of the pericardial space was performed to avoid thrombus formation within the pericardial space. For this purpose, the long sheath was retracted and two sheaths were introduced via seldinger technique into the epicardium using the same puncture hole. One sheath was used for flushing the epicardium with non-heparinized saline, while the other was used for drainage of the remaining blood and newly insufflated volume. This was repeated several times under continuous monitoring of hemodynamic parameters until aspirated fluid showed no more signs of bleeding with a serous consistence of aspirated fluidity. Also, blood gas analysis from epicardial blood was performed simultaneously to identify saturated- from unsaturated blood within the epicardial space, which may facilitate a potential location of rupture or perforation. Only if this work-up showed no sufficient effect in stabilization and bleeding still remained the final step was the consideration of open surgery as a final treatment option.

### Statistics and data collection

Continuous data were summarized as means ± standard deviations or as medians [25th and 75th percentiles] as appropriate. Categorical data were presented as N (%). Based on a logistic regression model (global test of no-regression) baseline variables were compared between the groups. Differences in procedural data were analyzed with the Wilcoxon-Mann Whitney test and in complications data using the Chi-square test or the Fisher’s exact test in case of small expected cell frequencies.

All *p*-values were two-sided and a *p*-value < 0.05 was considered significant. All calculations were performed with the statistical analysis software R (R Core Team 2018).

## Results

### Patients baseline data

A total of 211 patients (61.4 ± 15.6 years, 179 male; 84.8%) and 271 procedures were analyzed. An underlying structural heart disease was seen in 173/211 (82%) patients, whereas 38/211 (18%) patients showed structurally normal hearts. Epicardial access through inferior-oriented epicardial puncture (group 1) was performed in 152/211 patients (72%; 63.3 ± 15.1 years, 130male) in 189 procedures. Anterior-oriented epicardial access (group 2) was executed in 59/211 patients (28%; 59 ± 16.4 years, 49 male) in 82 procedures. Mean LV-EF was 46 ± 14 and similar in both analyzed groups (46 ± 13 vs 46 ± 15). Likewise, all baseline parameters did not differ significantly between group 1 and 2 (*p* = 0.857). Detailed baseline data are depicted in Table 1.

**Table 1:** Baseline patient characteristics

	All	Anterior-oriented puncture	Inferior-oriented puncture
*N* patients	211	59	152
*N* procedures	271	82	189
*N* male	179 (84.8%)	49 (83.1%)	130 (85.5%)
Mean age [years]	61.4 ± 15.6	59 ± 16.4	63.3 ± 15.1
Mean BMI [kg/m2]	26.3 [23.9,28.4]	26.4 [24.1,28.5]	26.3 [23.9,28.4]
Mean LV-EF [%]	46 ± 14	46 ± 13	46 ± 15
Mean LVEDD [mm]	56.8 ± 7.1	56.7 ± 7.1	56.8 ± 7.1
Structural heart disease	173 (82%)	50 (84.7%)	123 (80.9%)
iCMP	42 (24.3%)	9 (18%)	33 (26.8%)
dCMP	76 (43.9%)	27 (54%)	49 (39.8%)
ARVC	35 (20.2%)	6 (12%)	29 (23.6%)
Other	20 (11.6%)	8 (16%)	12 (9.8%)
Mean GFR [ml/min.]	67.1 ± 16.9	66.8 ± 17.6	67.3 ± 16.6
*N* pat. with previous heart surgery	6 (2.8%)	1 (1.7%)	5 (3.3%)
*N* pat. with aHTN	125 (59.2%)	38 (64.4%)	87 (57.2%)
*N* pat. with HLP	71 (33.6%)	22 (37.3%)	49 (32.2%)
*N* pat. with AFib	60 (28.4%)	22 (37.3%)	38 (25%)
*N* pat. with COPD	8 (3.8%)	1 (1.7%)	7 (4.6%)
*N* pat. with renal impairment	41 (19.4%)	14 (23.7%)	34 (17.8%)
*N* pat. with PAD	3 (1.4%)	1 (1.7%)	2 (1.3%)
*N* pat. on OAC/DAPT	71 (33.6%)	22 (37.3%)	49 (32.2%)
*N* pat. with procedures performed under general anesthesia with endotracheal intubation	20 (9.5%)	6 (10.2%)	14 (9.2%)
*N* proc. performed under general anesthesia with endotracheal intubation	24 (8.9%)	7 (8.5%)	17 (9%)
Test of no regression *p* = 0.857			

Metric data are summarized as means ± standard deviations or as medians [25th and 75th percentiles]. Categorical data are presented as N (%)

*pts.* patients, *iCMP* ischemic cardiomyopathy, *dCMP* dilative cardiomyopathy, *ARVC* arrhythmogenic right ventricular cardiomyopathy, *BMI* body mass index, *LV-EF* left ventricular ejection fraction, *LVEDD* left ventricular end diastolic diameter, *AFib* atrial fibrillation, *aHTN* arterial hypertension, *COPD* Chronic obstructive pulmonary disease, *HLP* Hyperlipidemia, *PAD* peripheral artery disease, *GFR* golmerular filtration rate, *OAC* oral anticoagulation, *NOAC* novel oral anticoagulant, *DAPT* dual antiplatelet therapy, *AAD* anti-arrhythmic drug

Test of no regression *p* = 0.857

### Procedural characteristics

The majority of procedures were performed under conscious sedation (247/271 proc., 91.1%), while 24/271 (8.9%) procedures were performed under general anesthesia with endotracheal-intubation (Table 1). Median procedure time in all cases was 222.5 min [160;280] with a median fluoroscopy time of 20 min [13;31] and a median fluoroscopy dosage of 2802 cGy*cm^2^ [1549;4779]. These parameters of procedure time, fluoroscopy time and fluoroscopy dosage, were significantly higher in group 1 (240 [180;300] vs. 175 [145;245], *p* < 0.001; 23 [16; 33] vs. 14 [10; 21], *p* < 0.001; 3294 [1803.8;5254.8] vs. 1760 [1302;3319], *p* < 0.001). Detailed data are presented in Tables 1, 2.

**Table 2:** Procedural parameters

	All	Anterior-oriented puncture	Inferior-oriented puncture	*P* value
N procedures	271	82	189	
Mean procedure duration (min.)	222.5 [160,280]	175 [145,245]	240 [180,300]	** < 0.001**
Mean fluoroscopy time (min.)	20 [13, 31]	14 [10, 21]	23 [16, 33]	** < 0.001**
Mean fluroscopy dosage (cGy*cm2)	2802 [1549;4779]	1760 [1302;3319]	3294 [1803.8;5254.8]	** < 0.001**

Metric data are summarized as medians [25th and 75th percentiles]. Categorical data are presented as *N* (%). *LL* Left lateral view, *AP* Anteroposterior view

### Periprocedural complications in the entire cohort

A total of 34 major periprocedural complications occurred in 271 procedures (12.6%) and 34 patients. Out of these, 23 complications (23/271; 8.5%) were directly related to the epicardial puncture. Pericardial tamponade was the most frequent complication, occurring in 24/271 procedures (8.9%) and in 15/271 procedures (5.5%) related to epicardial puncture. Data is shown in Table 3.

**Table 3:** Puncture-related complications

Total puncture-related complications—Anterior-oriented puncture	4/82 (4.9%)	*p * = 0.16
Pericardial tamponade	4 (4.9%)	
Intrahospital death	0 (0%)	
Total puncture-related complications—Inferior-oriented puncture	19/189 (10.1%)	
Pericardial tamponade	11 (5.8%)	
Abdominal organ puncture	5 (2.6%)	
Myocardial infarction	1 (0.5%)	
Pneumopericard	1 (0.5%)	
Pneumothorax	1 (0.5%)	
Intrahospital death	2 (1.1%)	

Metric data are summarized as medians [25th and 75th percentiles]. Categorical data are presented as *N* (%)

### Puncture-related complications in group 1—inferior epicardial approach (AP guided)

The incidence of puncture-related complications was 19/189 (10.1%) in group 1 with 14/189 (7.4%) cardiac and thoracic complications and 5/189 (2.6%) abdominal complications.

### Cardiac and thoracic complications

The most frequent complication in group 1 were pericardial tamponades (11/189; 5.8%). In 5/11 (45.5%) procedures pericardial tamponades were caused by an accidental puncture and consecutive misplacement of the sheath in the RV and in 4/5 patients (80%) emergent heart surgery was required during the process of the complication management described above. One of these patients died from septic shock with a pulmonary focus after prolonged mechanical ventilation. Massive epicardial thrombus formation within the epicardial space was found due to antagonization of heparin in one patient requiring heart surgery as well. The remaining 6/11 (54.5%) patients suffered from pericardial tamponade occurring directly after epicardial puncture with spontaneous termination of bleeding. The remaining three puncture-related complications in this group were an accidental puncture of the RCA, causing myocardial infarction and requirement of emergent cardiac surgery in one patient (1/189; 0.5%) and two thoracic complications. One case of iatrogenic pneumothorax was treated by the placement of a thoracic catheter which was removed after two days without any residues (1/189; 0.5%). An additional patient showed signs of a severe pneumopericardium (1/189; 0.5%). It was conservatively managed with a prolonged hospitalization (Table 3).

### Abdominal complications

The second most frequent complication in this group were abdominal organ perforations (5/189; 2.6%). Affected abdominal organs were the colon transversum and the liver in two patients respectively as well as gastric bleeding due to accidental puncture of a gastroepiploic artery in one patient. Patients with the accidental puncture of the liver were in congestive heart failure at the time of the procedure. In all patients with the accidental puncture of the liver and colon the long sheath was inserted via the abdominal organs into the epicardium. Onset of symptoms in these patients was > 6 h after cessation of the procedure and diagnosis was confirmed by computed tomography with consecutive emergency surgery. One patient with perforation of the colon transversum underwent surgical intervention with the requirement of a postoperative transversostoma. Another patient died after surgery from peritonitis and consecutive septic shock after surgery.

### Puncture-related complications in group 2—anterior epicardial approach (LAO 90° guided)

The incidence of severe complications related to the access within this group was 4/82 (4.9%). All complications documented within this cohort were pericardial tamponades, which were treated by pericardiocentesis. In none of these patients, an obvious puncture of the RV or abdominal organ perforations occurred and none of these four patients underwent emergent surgery. Patients in this group showed a lower tendency of puncture-related complications compared to group 1 (19/189 (10.1%) vs. 4/82 (4.9%); *p* = 0.16). All patients survived during hospital stay (Table 3).

### Major complications unrelated to epicardial puncture

**Fig. 3:**
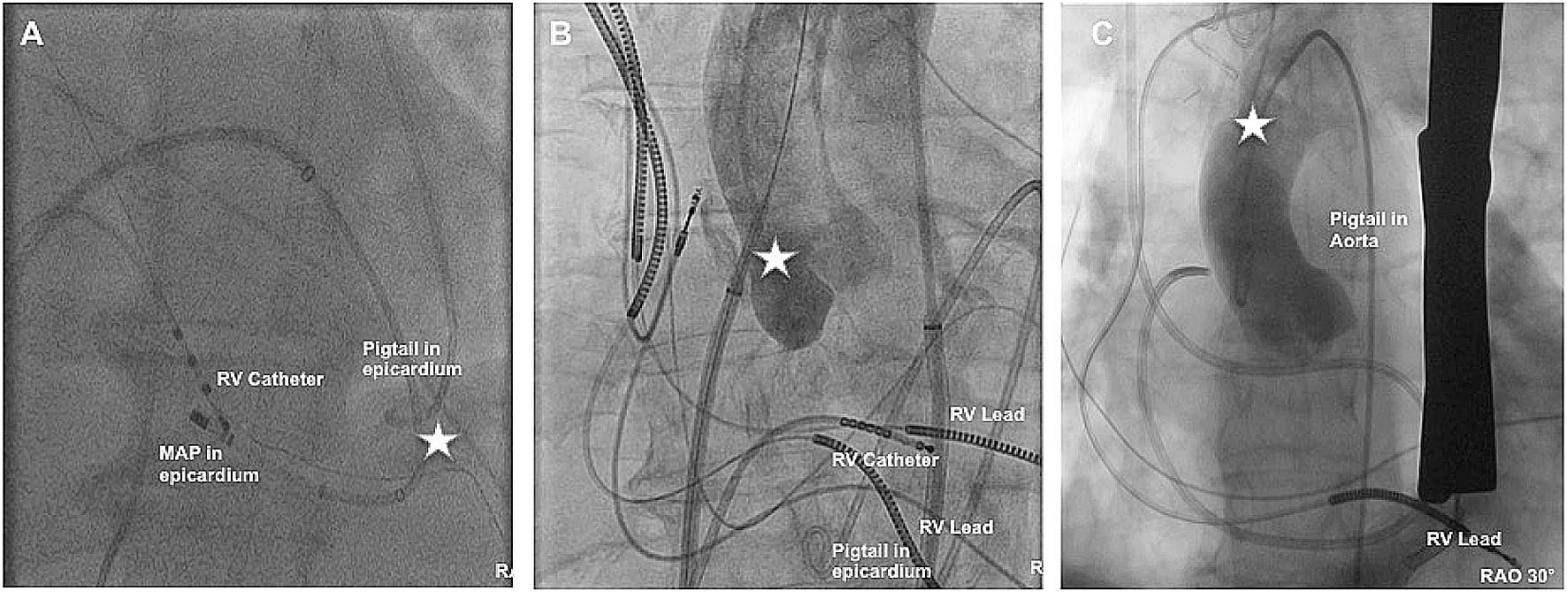
**a** Shows an apical LV perforation. The distal Ablation catheter is located epicardially. **b** Shows an accidental puncture of the aorta during transseptal puncture. The dilator is located in the aorta. **c** Shows an angiography after successful cardiac surgery due to perforation of the aorta ascendens via the distal sharp end of the SL1 Sheath. Star marks the perforation side. *RAO* Right anterior oblique, *RV* right ventricle, *MAP* Ablation catheter

Pericardial effusion or pericardial tamponades unrelated to epicardial puncture occurred in 9 patients. Causing mechanisms were inadequate transseptal puncture in 5/9 (55.6%), ventricular perforation during endocardial mapping/ablation in 3/9 (33.3%), and perforation of the coronary sinus with a diagnostic catheter in 1/9 patients (11.1%) (Fig. 3a, b). In one patient (0.4%) a perforation of the distal Aorta ascendens via the sharp distal end of the epicardial sheath was documented. This patient was referred to cardiac surgery due to continuous bleeding and survived without any sequelae (Fig. 3c). Acute pulmonary embolism occurred two days after procedure under paused anticoagulation due to a vascular groin complication in one patient and was managed conservatively.

### Minor complications unrelated to epicardial access and recurrence of VT

Severe symptoms of sterile pericarditis were reported in 29/271 (10.7%) cases. Conservative treatment using nonsteroid antiinflammatory drugs led to complete regression in all cases. The incidence did not differ significantly between the two groups (21/189 (11.1%) vs. 8/82 (9.8%) *p* = 0.906). Furthermore, a total of 6/271 (2.2%) minor groin complications occurred and were treated via monitoring by duplex sonography, bed rest and analgesia without any further treatment required.

Recurrence of the clinical VT during intrahospital follow-up occurred in 11/271 cases (4.1%) While 9/11 cases (81.8%) were treated conservatively through the administration of antiarrhythmic medication, short term re-ablation was performed in 2/11 cases (18.2%).

### Complication management of pericardial tamponade in the entire patient cohort

**Fig. 4:**
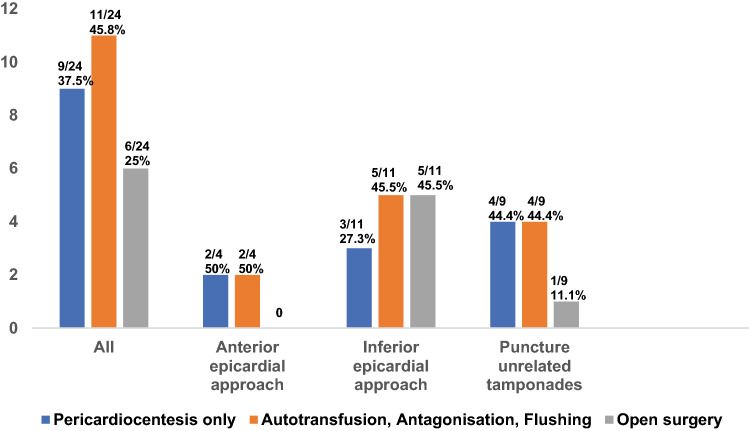
Different steps of pericardial tamponade management algorithm. Data are presented as *N* (%)

Pericardial tamponade was the most frequent major complication, occurring in 24/271 procedures (8.9%) overall, as well as in 15/271 procedures (5.5%) directly related to pericardial puncture. In all cases of pericardial tamponade, we applied a standardized algorithm of pericardiocentesis, followed by antagonization of heparin under continuous flushing and drainage of the pericardial space with simultaneous autotransfusion of the drained blood, escalated to surgical intervention in cases of no sufficient stabilization. In 9/24 (37.5%) patients sole pericardiocentesis was effectual to limit pericardial fluid-accumulation and patient stabilization. In case of prolonged pericardiocentesis with constant desistance of blood from the epicardial space, autotransfusion of aspirate and antagonization of heparin with simultaneous flushing and drainage of the pericardial space was performed in 11/24 patients (45.8%). In 2/24 patients (8.3%) insufficient conservative management led to surgical intervention. 4/24 patients (16.7%) patients were admitted to immediate surgical treatment without any further attempts of interventional management. Using this algorithm emergent surgery was avoided in 18/24 cases (75%; Fig. 4).

### Multiple procedures and pericardial adhesions

During the initial approach of epicardial access pericardial adhesions were found in 19/211 patients (9%). 6/19 (31.6%) patients with adhesions during the initial procedure had a history of previous cardiac surgery. History of severe perimyocarditis was identified as a probable pathogenesis in 1 case, while another patient had a history of cancer with consecutive chest radiation therapy. Therefore, the origin of observed pericardial adhesions during the baseline procedure remained idiopathic in 11/19 (57.9%) procedures. Nevertheless, successful epicardial mapping and ablation were performed in 6/19 (31.6%) patients in this subgroup.

Within the group of patients with observed pericardial adhesions during first epicardial access 3/19 (15.8%) patients had a second approach for epicardial access due to recurrent VA, while 1/19 (5.3%) had a third epicardial puncture respectively. Epicardial mapping and ablation was repeatedly successful in 1/3 (33.3%) patients during the second procedure but remained unsuccessful in the same patient during a third epicardial puncture.

**Fig. 5:**
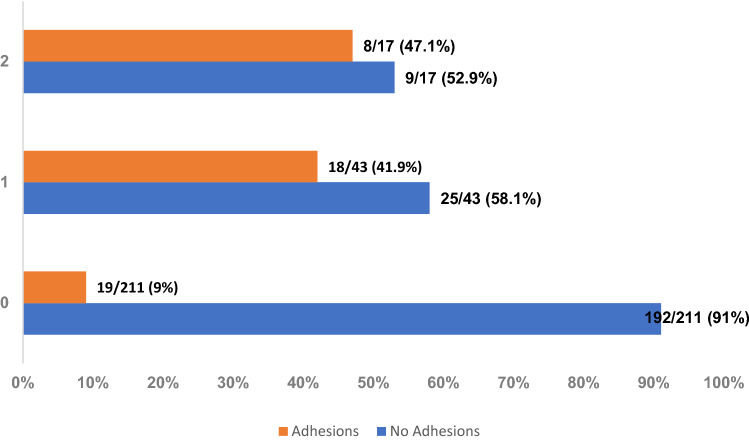
Incidence of Adhesions during epicardial ablation procedures. 0 = baseline procedure, 1 = first re-ablation with repetitive epicardial access, 2 = two or more ablation procedures with epicardial access. Data are presented as *N* (%)

Further analyzing the first epicardial re-puncture in patients who underwent a second approach for epicardial access, the incidence of pericardial adhesions increased to 18/43 (41.9%) and further rose to 47.1% (8/17) if patients had 2 or more epicardial re-punctures (19/211 vs. 18/43 vs. 8/17, *p* < 0.001) (Fig. 5). In total, procedures were limited due to pericardial adhesions in 45/271 procedures (16.6%) and complete epicardial map or ablation was not feasible in 36/45 procedures in patients with pericardial adhesions (80%).

No significant difference was observed in successful epicardial mapping and ablation in patients with epicardial adhesions between the groups (7/34 vs. 2/11, *p* = 0.79).

All patients with a history of cardiac surgery revealed pericardial adhesions during the initial epicardial puncture. 3/6 (50%) suffered from postinterventional complications. Successful inferior-oriented epicardial access was gained in 5/6 (83,3%) patients in this subgroup, while the only attempt for an anterior oriented access within this subgroup failed.

## Discussion

This study reveals our single-center experience of epicardial ablation of VA with regard to two different puncture techniques, complication management and the incidence of pericardial adhesions during the initial epicardial puncture, as well as after multiple epicardial approaches. Major findings of this study were (1) a higher incidence of major complications when performing an inferior oriented epicardial puncture compared to an anterior oriented epicardial puncture (10.1% vs 4.9%; *p* = 0.16); (2) adhesion within the epicardial space of 9% during first and up to 47% in patients with two or more epicardial procedures (3) prolonged procedure parameters when performing an inferior epicardial access.

### Correlation of complications within the two groups

Since Sosa et al. reported about their experience of successful percutaneous epicardial VT Ablation in 1997 in patients with Chagas disease [3] the number of epicardial VT ablation procedures increased rapidly. For more than one decade the AP-guided inferior oriented epicardial puncture technique has been the gold standard in approaching the epicardial space, even though the risk of potential access related complications were high and well known [4, 7]. Pericardial tamponade is still the most frequent complication in these procedures ranging from 6% up to 30% reported in the literature [7, 17, 20]. In our patient cohort, the overall incidence of pericardial bleeding complications was similar (8.9%). In addition, several reports on complications like abdominal organ perforations or collateral damages of coronary arteries have been published over the last years [7, 19, 21]. These complications were mainly observed while performing an AP-guided inferior-oriented puncture approach. In our cohort the incidence of major complications was 10.1% when performing an inferior epicardial approach. Comparison between the above-mentioned studies and our results are difficult since most reports did not differentiate explicitly between puncture-related and overall procedural complications. However, we also observed a not negligible number of severe abdominal collateral damages. In 5 patients (2.6%) perforations or accidental punctures of the liver, colon or gastric vessels occurred with the necessity of emergent surgery. We did not observe these complications at all when performing an anterior epicardial access approach with additional LAO 90° fluoroscopical guidance. There might be different reasons for this observation: First, during anterior epicardial access approach the needle is advanced right below the sternum after subxiphoidal puncture in a shallow angle. Therefore, it may enter the fibrous pericardium without penetrating the diaphragm with a lower risk of vessel injuries on the one hand, and without any other adjacent organs on the „pathway “ between the subxiphoid puncture side and pericardial access side (Fig. 2). Additionally, early imaging studies from the 1980s reported that pericardial fluid distribution in the supine position is mostly located anterior [22], which may facilitate a less difficult dry puncture performing an anterior epicardial puncture compared to a posterior access approach into the epicardial space. Nonetheless, the most common complication in our patient cohort was pericardial bleeding and consecutive tamponade. Interestingly, a previous study by Keramati et al. reported about no pericardial bleeding complications (> 80 ml blood) after LAO guided and anterior-oriented puncture and no emergent cardiac surgery or procedure-related mortality [13]. Even though we observed pericardial bleeding in 4 patients (4.9%) in the anterior epicardial group, we also did not experience any need for emergent cardiac surgery or procedure-related mortality. However, patients in the study mentioned above suffered from ARVC in a certain proportion and may have had less comorbidities compared to our patient population. The authors also state that they did not perform endocardial mapping in all patients and consecutively did not heparinize them.

### Complication prevention and management

Besides the observation of specific complications arising from the two epicardial puncture techniques, some general considerations seem to be relevant, that may help avoiding complications. A preprocedural abdominal sonography can be a helpful tool to discriminate the dimensions of the liver, especially in patients with acute decompensation of heart failure. In these cases, a more lateral puncture site, guided by previous sonography findings, might be reasonable.

In patients with iatrogenic injury of abdominal organs, symptoms like abdominal pain began on average 6–8 h after cessation of the procedure. This underlines the necessity of careful monitoring, even in patients initially stable and asymptomatic after the procedure. Abdominal sonography for organ injury and free fluids can be a useful tool for efficient bedside examination and early diagnosis if abdominal organ injury is suspected, while an abdominal CT-scan represents the most accurate and sensitive diagnostic instrument. Another important issue seems to be the risk of accidental puncture of the RV, which does not necessarily result in severe bleeding complications if the sheath has not been advanced into the RV completely. To overcome the risk of bleeding complications after accidental puncture of the RV, several groups analyzed different techniques and tools [7, 23]. One important tool can be the use of a micropuncture needle (needle in needle technique) [9, 24]. A LAO guided anterior-oriented puncture technique performed with a micropuncture needle might be a beneficial combination to prevent RV puncture, even though radiation exposure is particularly high for the operator when using a LAO 90° angulation.

An aggravating factor in this context might be anticoagulation management. The risk of severe bleeding complications can overweigh the risk of thrombus formation. Therefore, if anticoagulation is needed, the possibility of administration after completed epicardial puncture should always be considered thoroughly and on an individual base.

Furthermore, in situations of severe bleeding events a standardized complication management protocol can be essential and lifesaving. The majority of patients suffering from bleeding complications did not require complex open-heart surgery, as bleeding terminated while the patients were stabilized using our coordinated algorithm. One patient of our cohort underwent emergent cardiac surgery due to massive thrombus formation within the pericardial space after antagonization of heparin with protamine and additional administration of fibrinogen. Therefore, we would recommend a careful administration of sole protamine performed only under simultaneous flushing of the epicardial space (with non-heparinized saline) and consecutive aspiration. However, this complication algorithm has to be evaluated prospectively.

Simultaneous endocardial mapping was performed during most of the epicardial ablation procedures. Inappropriate mapping and/or transseptal puncture during endocardial mapping, as well as coronary perforation are possible additional causes of pericardial tamponade. In these situations, blood-gas analysis to identify saturated from unsaturated blood within the epicardial space may help differentiating a RV perforation from a LA or left ventricle perforation.

Another important factor in complication prevention is the operator experience in these high risk procedures. Even though we observed a reduction of complications over the study time and some operators at our institution gained more experience, it must be taken into account that there was fluctuation among the operators over the period of almost fifteen years and the analyzed procedures were carried out by different interventionalists. Due to this fluctuation and the high number of different invasive electrophysiologists performing epicardial VT ablation procedures at our high-volume center, the influence of the experience of the individual operator on the overall complication rate appears very small compared to the puncture technique in general.

### Epicardial access after multiple procedures and adhesions

Even after reducing the risk of major complications with all available techniques and tools, many factors, like comorbidities and patient selection influence procedural safety. Limited access to the epicardium due to adhesions can also increase the risk of complications [14]. Adhesions after prior cardiac surgery are almost universal and the only attempt for an anterior oriented access within this subgroup failed, most likely since post-surgery adhesions usually concern the anterior or lateral pericardium. Less is known about the prevalence of adhesions in patients without previous cardiac surgery or a history of perimyocarditis. A recent study reported about 155 epicardial procedures with 8% adhesions after the first and 13% after the second epicardial ablation [15]. Our data are in line with the findings concerning de novo adhesions, but the incidence of adhesions in repeated epicardial procedures was distinctly higher in our cohort. However, the periprocedural risk increases when having adhesions and may require blunt dissection of adhesions with a steerable sheath or even a surgical creation of a pericardial window and manual dissection of the adhesions by the hand of the cardiac surgeon. A blunt dissection of adhesions with a steerable sheath increases the risk of bleeding complications in theory but has not been investigated systematically so far. Taking all this into consideration a critical use-risk analysis should be performed prior to every epicardial ablation, especially before repeated approach for epicardial access in the same patient.

### Clinical implication

This is, to the best of our knowledge, the first study analyzing and comparing the two most commonly used puncture techniques in a large patient cohort from a high volume center for VT ablation. It is important to state that the gain of information about procedural complications, their pathomechanisms and management is not only important for further performance of safe epicardial electrophysiological procedures, but also for every cardiologist in terms of acute complication management and performance of safe pericardiocentesis. However, taking all these aspects into account, the availability of surgical backup in all procedures with epicardial access, as currently recommended by the recent consensus statement of the HRS/EHRA/APHRS/LAHRS should be the gold standard [25].

### Limitations

This was a retrospective study with its typical limitations. Our analysis contains only cases of intrahospital complications and mortality without further follow-up. Further studies are necessary to prospectively evaluate these results. More importantly, AP guided inferior-oriented puncture was initially used and changed to LAO guided anterior-oriented puncture after gaining more experience and knowledge about epicardial puncture and potential complications. Nevertheless, our analysis is to our knowledge the first study in a large patient cohort comparing these two epicardial puncture techniques.

## Conclusions

The risk of major complications in epicardial procedures is high, but might be reduced using an anterior-oriented epicardial access. Repeat epicardial access increases the incidence of pericardial adhesions significantly.

## Data Availability

Not applicable.
